# Wheat Bran Proteins as a Functional Ingredient in Plant‐Based Mayonnaise

**DOI:** 10.1002/fsn3.71956

**Published:** 2026-05-31

**Authors:** Varshini Krishnamoorthy, Abinaya Arunachalam, Jan Krijgsheld, Gert‐Jan W. Euverink

**Affiliations:** ^1^ Faculty of Science and Engineering Products and Processes for Biotechnology, Engineering and Technology Institute Groningen, University of Groningen Groningen the Netherlands; ^2^ Polymer Science Zernike Institute for Advanced Materials, University of Groningen Groningen the Netherlands; ^3^ Research and Development, Royal Koopmans Leeuwarden the Netherlands

**Keywords:** Herschel–Bulkley model, plant‐based mayonnaise, rheology, wheat bran protein

## Abstract

Mayonnaise is an oil‐in‐water emulsion and traditionally contains egg yolk as an emulsifier. The growing trend of veganism and vegetarianism has instilled the need to replace egg yolk with plant‐based protein to formulate mayonnaise analogues. The current study was focused on substituting egg yolk with Osborne fractionated wheat bran proteins (albumin, globulin, and glutelin). The pH, stability, color, and rheological properties of the prepared vegan emulsion were studied and compared with traditional mayonnaise containing egg yolk (control). The pH and emulsion stability of albumin mayonnaise were comparable to the control, indicating its effectiveness as an emulsifying agent. A significant difference was observed in the color measurements of L*, a*, and b* values for vegan mayonnaise, resulting in a brownish formulation. The rheological results showed pseudo‐plastic, thixotropic behavior, and the data were well described by the Herschel–Bulkley model. Mayonnaise formulated with albumin presented a higher yield stress (*τ*_0_) than globulin and control mayonnaise. The flow index (*n*) of all three samples exhibited shear‐thinning, non‐Newtonian (*n* < 1) fluid behavior. Overall, the results demonstrate that the functional performance of mayonnaise is strongly influenced by the protein fraction used. This study highlights the potential application of wheat bran protein fractions in plant‐based mayonnaise as a viable and sustainable alternative to traditional egg‐based mayonnaise.

## Graphical abstract

Wheat bran proteins (albumin, globulin, and glutelin) were extracted through Osborne fractionation and incorporated into mayonnaise formulations as plant‐based emulsifiers. Among the fractions, albumin‐based mayonnaise demonstrated stable emulsion properties at acidic pH (4.6) and a texture comparable to traditional mayonnaise, highlighting its potential as a sustainable vegan alternative.

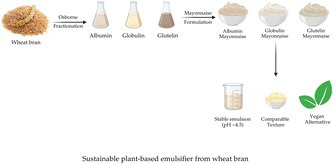

## 1 Introduction

An oil‐in‐water emulsion is characterized by an oil phase dispersed in an aqueous phase, stabilized by an emulsifier. The lipophilic and the hydrophilic ends of the emulsifier molecule hold the fat and water together to form a stable emulsion. Traditional oil‐in‐water emulsions, such as mayonnaise, contain 70%–80% oil and are among the most popular high‐calorie condiment products. Mayonnaise, a semi‐solid formulation, is prepared by mixing oil, water, salt, sugar, vinegar, flavoring agent (such as mustard flour), and emulsion stabilizers (often egg yolk). The thermodynamically unstable oil‐in‐water emulsion is kinetically stabilized by optimizing the processing parameters (such as temperature, blending duration, and ratio), the volume fractions of the continuous and dispersed phases, and the viscosity of the continuous phase (Meybodi et al. 2014). The quality of mayonnaise is often determined by interactions between ingredients and oil droplets as they directly affect the lubrication, perceived texture, and flavor release (Taslikh et al. 2022). For example, an increase in adhesiveness and firmness is observed when the oil droplet size decreases, which in turn affects the taste or flavor of mayonnaise (Olsson et al. 2018). On the other hand, an increase in oil droplet diameter leads to creaming and phase separation, affecting the storage time (Taslikh et al. 2022).

Among mayonnaise ingredients, egg yolk, an emulsifier/emulsion stabilizer, is a crucial ingredient that confers stability to the condiment. Although egg yolk possesses excellent emulsion properties, the increased levels of cholesterol and saturated fats lead to health conditions such as obesity, cardiovascular diseases, and high blood pressure (Mozaffarian 2016; Yildirim et al. 2016). Furthermore, extensive use of animal protein will result in intensive animal husbandry, thus impacting the ecosystem (Liu et al. 2018). Using plant‐based alternatives not only reduces the risk of health problems but also reduces the cost of procurement and processing, increases microbial stability, and lowers the carbon footprint in comparison to animal protein (Coda et al. 2017; Lan et al. 2018; Riscardo et al. 2003). Plant‐based proteins (such as wheat, soy, pea, and faba bean) have been used as emulsifiers in food systems due to their ability to stabilize the oil–water interphase and reduce the interfacial tension between hydrophilic and lipophilic groups (Raymundo et al. 2002). Over the years, many studies have reported partial or complete replacement of egg yolk with plant‐based alternatives such as faba bean protein, pea pod powder, soybean protein, wheat gluten, and soy milk, which mimic the creaminess, texture, and mouthfeel of the traditional mayonnaise (Diftis et al. 2005; Kumar et al. 2021; Liu et al. 2018; Nikzade et al. 2012; Ouraji et al. 2020; Rudra et al. 2020). Wheat bran proteins have good emulsifying and water‐binding properties, thereby improving the stability and texture of mayonnaise (Idris et al. 2003). Therefore, the addition of novel ingredients such as wheat bran protein to mayonnaise formulation is promising, although it is also necessary to evaluate their rheological properties. Evaluating the rheological properties of the emulsion system is crucial in determining the formulation, quality control, and process conditions. Further, mayonnaise's texture and rheological properties are often known to influence the mouthfeel (Izidoro et al. 2008; Liu et al. 2007; Schädle et al. 2022). Mayonnaise shows pseudoplastic behavior with a flow threshold, thixotropy, and exhibits time‐dependent characteristics (Guilmineau and Kulozik 2007; Paredes et al. 1989; Peressini et al. 1998). The complex rheology of mayonnaise has been studied using steady shear and oscillatory shear measurements. For steady shear measurements, the flow properties of mayonnaise and salad dressings are described by Herschel–Bulkley, power law, Carreau, and Casson models (Bistany and Kokini 1983; Guilmineau and Kulozik 2007; Liu et al. 2007; Paredes et al. 1989).

Wheat bran is a by‐product obtained from wheat milling. The by‐product is rich in bioactive compounds and contains 13%–18% protein, comprising essential amino acids (such as lysine, histidine, methionine, and valine) (Uttam et al. 2023). Proteins from wheat bran have been reported to have good emulsifying, foaming, and solubility properties and are suggested as plant‐based alternatives for animal protein (Idris et al. 2003). Replacing egg yolk with wheat bran protein would be beneficial in overcoming adverse health conditions by lowering cholesterol levels. Furthermore, this would also address the interests of the growing vegan population and, in turn, would be an effective strategy to promote a sustainable food system (Van Der Goot et al. 2016).

To date, there is no published data in the literature on the application of wheat bran protein fractions as a functional ingredient to completely replace egg yolk. The current study is aimed at sequentially fractionating wheat bran proteins based on their solubility in water, salt, and alkali into albumin, globulin, and glutelin, respectively, to form a stable emulsion system in a mayonnaise formulation (Sardari et al. 2019). The rheological properties and tribological measurements of vegan mayonnaises are determined. The conclusion of this study could be advantageous for the condiment industry in manufacturing plant‐based mayonnaise to substitute/replace traditional mayonnaise with a similar texture, mouthfeel, and sensory profile.

## 2 Materials and Methods

### 2.1 Chemicals

The main ingredients for the emulsion formulation, such as sunflower oil, sugar, table salt (sodium chloride), and mustard flour, were purchased from the local supermarket in The Netherlands. Wheat bran for protein extraction was supplied by Koopmans Meel B.V., Leeuwarden, The Netherlands. Egg yolk (Catalogue No. E0625) and other chemicals were purchased from Sigma‐Aldrich (Sigma Chemical Co., St Louis, MO). Chemicals used were of analytical grade.

### 2.2 Reducing Particle Size and Defatting of Wheat Bran

The particle size of wheat bran was reduced by grinding the bran in a Retsch jaw crusher BB 400 (Haan, Germany) to a particle size of 700 μm. Wheat bran was defatted according to Sahu et al. (2021) with some modifications. The wheat bran was defatted with hexane at a 1:4 ratio and was constantly stirred in a fume hood at room temperature for 90 min. The suspension was filtered, and defatting was repeated three times. The residue was dried overnight in a fume hood for further extractions.

### 2.3 Osborne Fractionation of Wheat Bran Proteins and Membrane Filtration

The protein extraction from wheat bran and membrane filtration were carried out as described by Sardari et al. (2019).

### 2.4 Emulsion Preparation

The recipe for mayonnaise contained the following ingredients in percentage (w/w): water 17.1, salt 0.8, sugar 4, mustard flour 0.5, vinegar 7.5, oil 60, and wheat bran protein fraction 10. The mayonnaise samples were prepared by completely replacing egg yolk with wheat bran protein fractions. The vegan mayonnaise containing wheat bran protein fraction as an emulsifier was compared with the mayonnaise containing egg yolk. Mayonnaise was prepared in a three‐step process. In the first step, dry ingredients, namely, salt, sugar, mustard flour, and wheat bran protein fractions, were combined, followed by the addition of water. The mixture was blended using a commercial hand frother for 2 min, and half the volume of oil was added drop‐wise using a syringe to the mixture with constant blending. The mixture was mixed for 2–3 min. In the second step, vinegar was gradually blended in. In the third step, the remaining oil was added drop‐wise and blended. The mayonnaise samples were prepared at 22°C ± 2°C and stored at 4°C for further analysis.

### 2.5 Stability Test

The physical stability of mayonnaise was evaluated according to Yildirim et al. (2016) with modifications. Samples (5 g) were taken in pre‐weighed falcon tubes and centrifuged at 5000 rpm for 30 min. The supernatant was discarded, and the weight of the precipitate was measured. The physical stability of the prepared emulsion was determined based on the emulsified layer weight to the total sample weight:

(1) $$\text{Stability}\;\left(\%\right)=\frac{F_{1}}{F_{0}}\times 100$$

where *F*_0_ and *F*_1_ denotes the weight of the sample before and after centrifugation, respectively.

The thermal stability of the samples was studied as described by Rahmati et al. (2014) with modifications. Briefly, 5 g of the prepared emulsions were taken in falcon tubes and incubated at 80°C for 30 min. The falcon tubes were centrifuged at 5000 rpm for 30 min and the thermal stability was calculated using the equation above.

### 2.6 Moisture Analysis and pH

The moisture content of the prepared emulsions was determined using a KERN DLT‐N moisture analyzer (Kern & Sohn GmbH, Balingen, Germany). The pH of the samples was measured, without dilution, using a VOS‐70002 pH meter (VOS instrumenten, Zaltbommel, The Netherlands).

### 2.7 Color Measurements

The color of the emulsion was measured using PCE‐CSM2 (PCE Instruments, Enschede, The Netherlands). The color parameters were determined by the CIEL L*a*b system (Flamminii et al. 2020). The parameters include L* lightness (0—black, 100 white), a* (−a* green, +a* red), and b* (−b* blue, +b* yellow), C*_ab_ (saturation), and h^o^_ab_ (hue angle). The measurements were conducted at 20°C ± 0.5°C under constant lighting conditions. The average of triplicate measurements for each sample was used for analysis.

### 2.8 Microscope Imaging

A drop of each mayonnaise sample was placed on a glass slide and covered with a coverslip. The microstructure of the emulsion was evaluated at a magnification of 20× using an Olympus CX41 Microscope (Pennsylvania, United States of America).

### 2.9 Rheological Properties

The rheological properties of vegan mayonnaise were measured using a HAAKE Modular Advance Rheometer System (III) (MARS) (ThermoFisher Scientific, Massachusetts, USA) equipped with a cone plate geometry (*d* = 20 mm) and temperature control system. Flow curves were determined at 20°C ± 0.5°C in three steps. The shear rate was increased linearly (0.5–300 s^−1^), held at 300 s^−1^, and linearly decreased to 0.5 s^−1^. The average of triplicate measurements for each sample was used for analysis.

The Herschel–Bulkley model for non‐Newtonian fluids was used to characterize the rheological properties of the prepared emulsion (Schädle et al. 2022):

(2) $$\tau =\tau_{0}+K\;{ϒ}^{n}$$

where τ is the shear stress (Pa); *τ*_0_ is the yield stress (Pa); ϒ is the shear rate (s^−1^); *n* is the flow index (−); and *K* is the consistency index (Pa.s^n^).

The Kokini Oral Shear Stress (OSS) model was used to theoretically estimate the in‐mouth viscosity of the mayonnaise sample. The Kokini OSS is calculated using the parameters from the Herschel–Bulkley model and determined using the equation (Cook et al. 2003):

(3) $$\tau =\tau_{0}+{\mathrm{Kv}}^{n}\;{\left(\frac{1}{h_{0}^{\left(n+1\right)/n}}+{\left(\frac{F}{R^{n+3}}\times \frac{n+3}{2\mathrm{\pi K}}\right)}^{1/n}\times \frac{\left(n+1\right)t}{2n+1}\right)}^{n^{2}/\left(n+1\right)}$$

where *ν* is the velocity of the tongue (2 cm s^−1^); *F* is the normal force (1 N), *R* is the radius of the plug (2.5 cm), *t* is time (1 s); *h*_0_ is the initial plug height (0.2 cm).

### 2.10 Probe Tack Test

Probe tack tests were performed on an AntonPaar MCR302e rheometer equipped with a flat sand‐blasted stainless‐steel probe with a diameter of 10 mm (PP10/S). The bottom plate was maintained at a constant temperature of 20°C, following which samples were loaded onto the rheometer. The probe was lowered until the sample thickness reached 150 μm. After allowing 1 min for the sample to make adequate contact with the probe, it was retracted at a speed of 100 μm s^−1^. The normal force was recorded as a function of gap width, which was then converted into stress and strain using the following equations:

(4) $$\text{Stress}\;\left(\sigma \right)=\frac{F}{A₀}$$

(5) $$\text{Strain}\;\left(\varepsilon \right)=\frac{h-h₀}{h₀}$$

Work of adhesion (*W*_adh_) was subsequently calculated as the integral of the stress versus strain curve using:

(6) $$\text{Work of adhesion}\;\left(W_{\mathrm{adh}}\right)=h₀\int_{0}^{\varepsilon }\sigma \;\mathrm{d\varepsilon}$$

where *F* is the measured normal force; *A*_0_ is the area of the probe; *h* is the position of the probe; and *h*_0_ is the initial gap = 150 μm.

The average of triplicate measurements for each sample was used for analysis.

### 2.11 Statistical Analysis

All experiments were conducted in triplicate (*n* = 3), and data are presented as mean ± standard deviation (SD). This study was exploratory and descriptive in nature; therefore, no inferential statistical analyses were performed to assess significant differences between samples.

Data analysis and visualization were performed using OriginPro 2024 and Microsoft Excel.

## 3 Results and Discussion

### 3.1 Extraction of Wheat Bran Proteins

The total protein extraction yield of 58.8% was obtained based on defatted wheat bran, with albumin, globulin, and glutelin accounting for 30.1%, 5.6%, and 23.0%, respectively.

### 3.2 Physical Appearance of Mayonnaise

**FIGURE 1:**
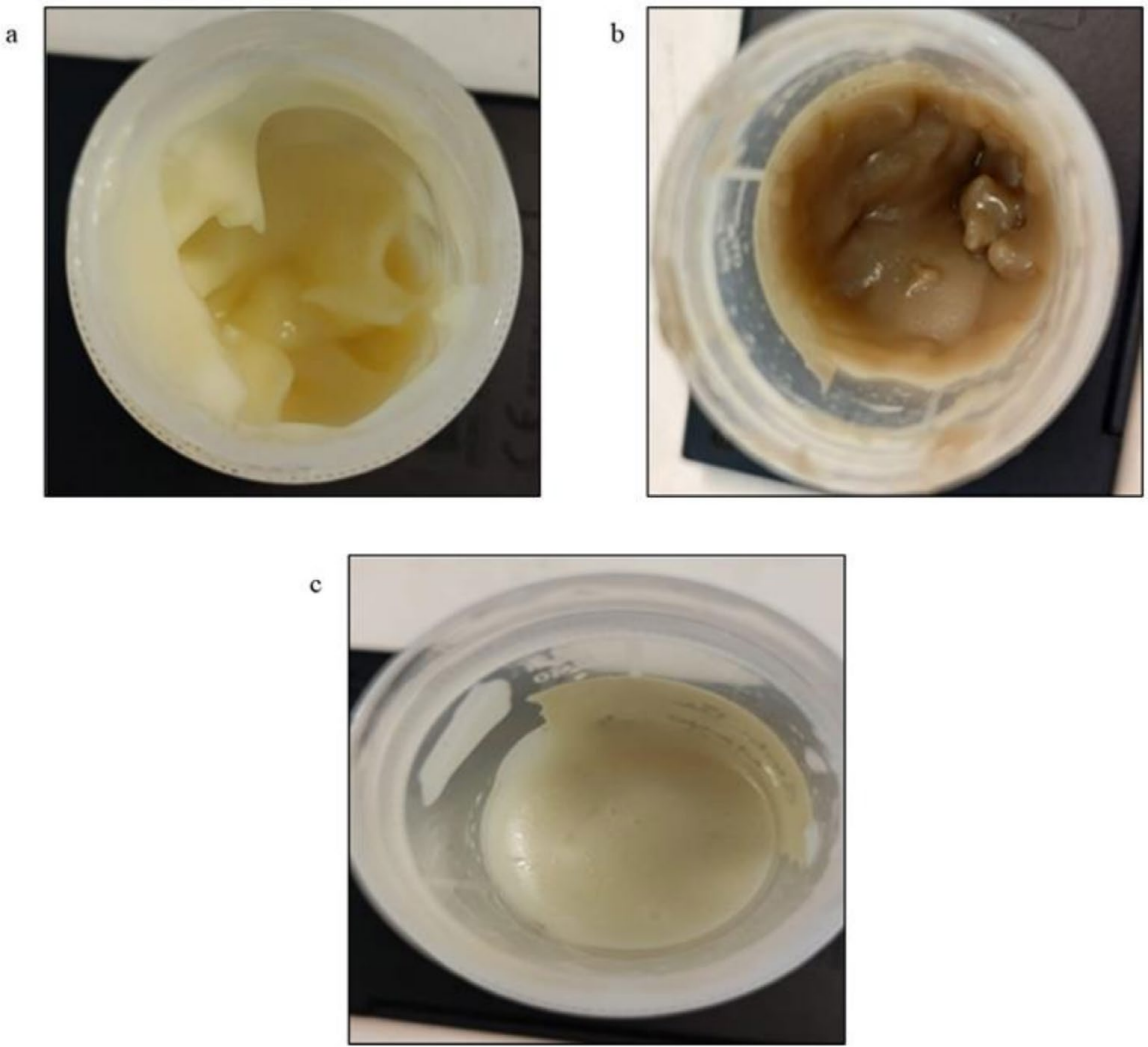
Physical appearance of mayonnaise (a) egg yolk; (b) albumin; (c) globulin.

Figure 1 shows the physical appearance of mayonnaise made with egg yolk, albumin, and globulin. The control mayonnaise shows a pale‐yellow coloration. The albumin mayonnaise exhibited a brown coloration, suggesting the presence of co‐extracted phenolic compounds and flavonoids, and enzymatic browning (Matus‐Cádiz et al. 2008). The pale white color of globulin mayonnaise can be due to limited binding of phenolic compounds and minimal co‐extraction of pigments during salt‐based extraction (Jimenez‐Pulido et al. 2022).

### 3.3 Stability

The shelf‐life of condiment products is often determined by the physical stability of the emulsion under centrifugal force and its thermal stability at elevated temperature. Emulsion stability is affected by an increase in the size of the oil droplet and uniformity. Additionally, temperature difference, applied pressure, type, and concentration of emulsifier play a role in destabilizing the emulsion (Harrison and Cunningham 1985).

**FIGURE 2:**
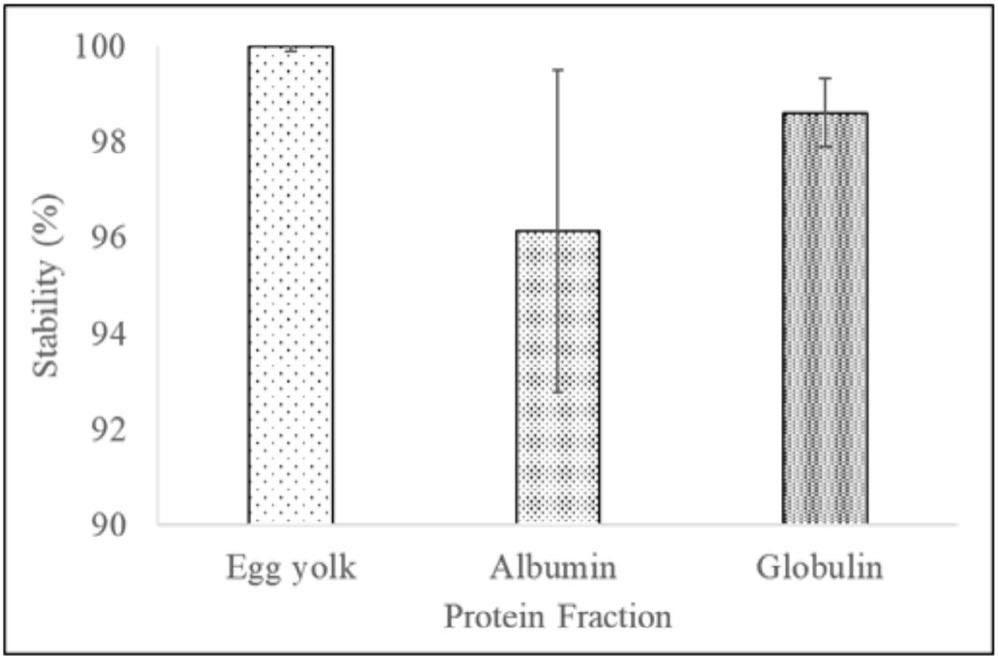
Physical stability of vegan mayonnaise compared to the control. Data are presented as mean ± SD (*n* = 3).

Figure 2 shows the stability of vegan mayonnaise samples compared to the control. The physical stability of albumin (96.1% ± 3.4%) and globulin (98.6% ± 0.7%) mayonnaise was comparable with that of the control (100% ± 0.1%). No significant phase separation was observed. Similar findings have been reported in mayonnaise studies with defatted soy flour (100%), soy flour (95.7%–98.3%), and pea pod powder (> 99.86%) (Lee et al. 2024; Rudra et al. 2020). Egg‐free mayonnaise with *Cicer arietinum* (chickpea), *Vicia faba* (faba bean), and *Lens culinarius* (yellow split lentils) protein showed no separation. A 100% aquafaba mayonnaise reported an emulsion stability of 80.62% (Ozcan et al. 2023). Mayonnaise with soy milk and stabilizers such as mono‐ & diglycerides emulsifier, guar gum, and xanthan gum showed an emulsion stability of 65.7%, 93.0%, and 97.8%, respectively. However, the stabilizers used in combinations reported an increase in the emulsion stability (74.5%–97.6%) (Nikzade et al. 2012).

The high stability of mayonnaise in the current study can be attributed to the presence of microstructure, characterized by a relatively uniform distribution of oil droplets, as evidenced by the microscopic analysis (Figure 4). Furthermore, the proteins prevent the oil droplets from coalescing by forming a barrier at the interface (Berton‐Carabin et al. 2018; Harrison and Cunningham 1985).

Thermal stability is a critical parameter for evaluating the shelf‐life and storage conditions of mayonnaise as well as its suitability for use on food items. The decrease in stability is a consequence of large oil droplets, a decrease in viscosity, and the movement of emulsion droplets in the aqueous phase. The presence of protein or polysaccharides helps in preventing coagulation, increases viscosity, and limits the movement of oil droplets, in turn, enhancing the stability of the emulsion system (Herald et al. 2009; Mirsadeghi Darabi et al. 2022).

**FIGURE 3:**
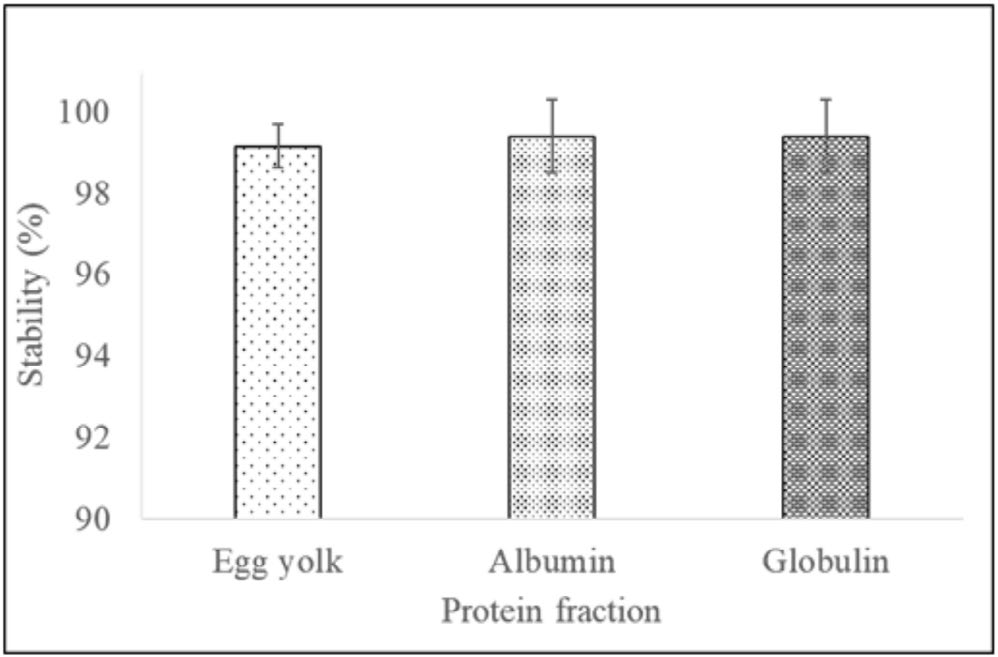
Thermal stability of vegan mayonnaise compared to the control. Data are presented as mean ± SD (*n* = 3).

The thermal stability of vegan mayonnaise relative to the control is shown in Figure 3. Albumin and globulin mayonnaise exhibited a thermal stability of 99.4% ± 0.9%. These results were comparable to the thermal stability of the control (99.2% ± 0.5%).

A study by Karshenas et al. (2018) on mayonnaise with sesame, peanut, and sesame‐peanut meal milk reported thermal stability at 100%. Mayonnaise with faba bean, defatted soy flour, and soy flour showed a thermal stability index of 98.77% ± 0.01%, 97.7%–99.5%, and 93.1%–96.8%, respectively (Ouraji et al. 2020; Lee et al. 2024). These results are comparatively lower than the stability of mayonnaise made with albumin and globulin from wheat bran reported in the current study. A study by Ghoush et al. (2008) reported a significant decrease in the stability of mayonnaise prepared with wheat protein and iota‐carrageenan. The decrease in stability was due to rapid flocculation and coalescence of small droplets as the storage temperature increased.

Overall, the emulsion stability (physical and thermal) of vegan mayonnaise was at par with the control. This can be attributed to the higher concentration of protein, exhibiting increased hydrophobicity, which enhances adsorption at the droplet surface (Lee et al. 2024). Second, a study by Takeda et al. (2001) suggests the viscoelastic protein film surrounds the oil droplets, prevents coalescence, and enhances the stability of the emulsion system. Thus, this confirms that wheat bran protein fractions can effectively serve as an emulsifier to stabilize an oil‐in‐water emulsion system.

### 3.4 pH and Moisture Content

The pH influences microbial growth, sour flavor, viscosity, and shelf‐life. Commercial mayonnaise typically has a pH in the range of 3–4.5. The pH measurement of vegan mayonnaise is presented in Table 1. The pH of globulin mayonnaise was close to neutrality, which is prone to microbial contamination. This would, in turn, affect the sensory profile and shelf‐life of mayonnaise. Further, the pH of albumin mayonnaise was comparable to that of the control and aligns with other plant protein‐based mayonnaise mentioned in the literature.

**TABLE 1:** Comparison of pH values and moisture content of vegan mayonnaise and control.

Formulation	pH	Moisture content (%)
Egg yolk (control)	4.3 ± 0.0	24.0 ± 2.7
Albumin	4.6 ± 0.5	21.0 ± 3.6
Globulin	6.1 ± 0.1	11.8 ± 0.2

*Note:* Data are presented as mean ± SD (*n* = 3).

The long‐term stability of the condiment depends on the moisture content. The control and albumin mayonnaise exhibited similar moisture content (Table 1). The moisture content of full‐fat mayonnaise was studied by Liu et al. (2007) was 13.74%, while the moisture content of mayonnaise samples containing different fat mimetics ranged from 47.5% to 54.4%. Mayonnaise with low‐moisture content is preferred as it enhances stability, increases viscosity, and reduces phase separation (Depree and Savage 2001).

### 3.5 Color Measurements

Color measurements are crucial as they are the primary sensory perception influencing consumer acceptance. Mayonnaise is a typical pale‐yellow emulsion, and the color originates from xanthophylls, carotene, cryptoxanthin, and lutein present in egg yolk (Herald et al. 2009). The lightness (L*), redness (a*), and yellowness (b*) of control and vegan mayonnaise are shown in Table 2. The L* of albumin and globulin mayonnaise was significantly lower than the control, while the a* was higher. Similar results with decreased L* and increased a* were observed for chickpea protein isolate (Kaur and Singh 2007). Further, studies have reported a decrease in L* of mayonnaise when substituted with plant‐based emulsifiers (Karshenas et al. 2018; Ozcan et al. 2023; Rahmati et al. 2014). A variation in redness could be due to protein‐polyphenol, protein‐mineral, and/or protein‐pigment interactions (Alu'datt et al. 2017). Other factors affecting color measurements include fat content, additives, spices with coloring effect (such as turmeric), and droplet size (an increase in droplet size restricts light scattering) (Flamminii et al. 2020).

**TABLE 2:** Color measurements of vegan mayonnaise and control.

Formulation	L*	a*	b*
Egg yolk (control)	72.8 ± 1.0	−1.4 ± 0.2	18.6 ± 2.6
Albumin	36.8 ± 1.6	9.3 ± 0.1	20.9 ± 1.9
Globulin	51.6 ± 1.2	7.7 ± 1.9	17.8 ± 3.5

*Note:* Data are presented as mean ± SD (*n* = 3).

### 3.6 Microscopic Imaging

**FIGURE 4:**
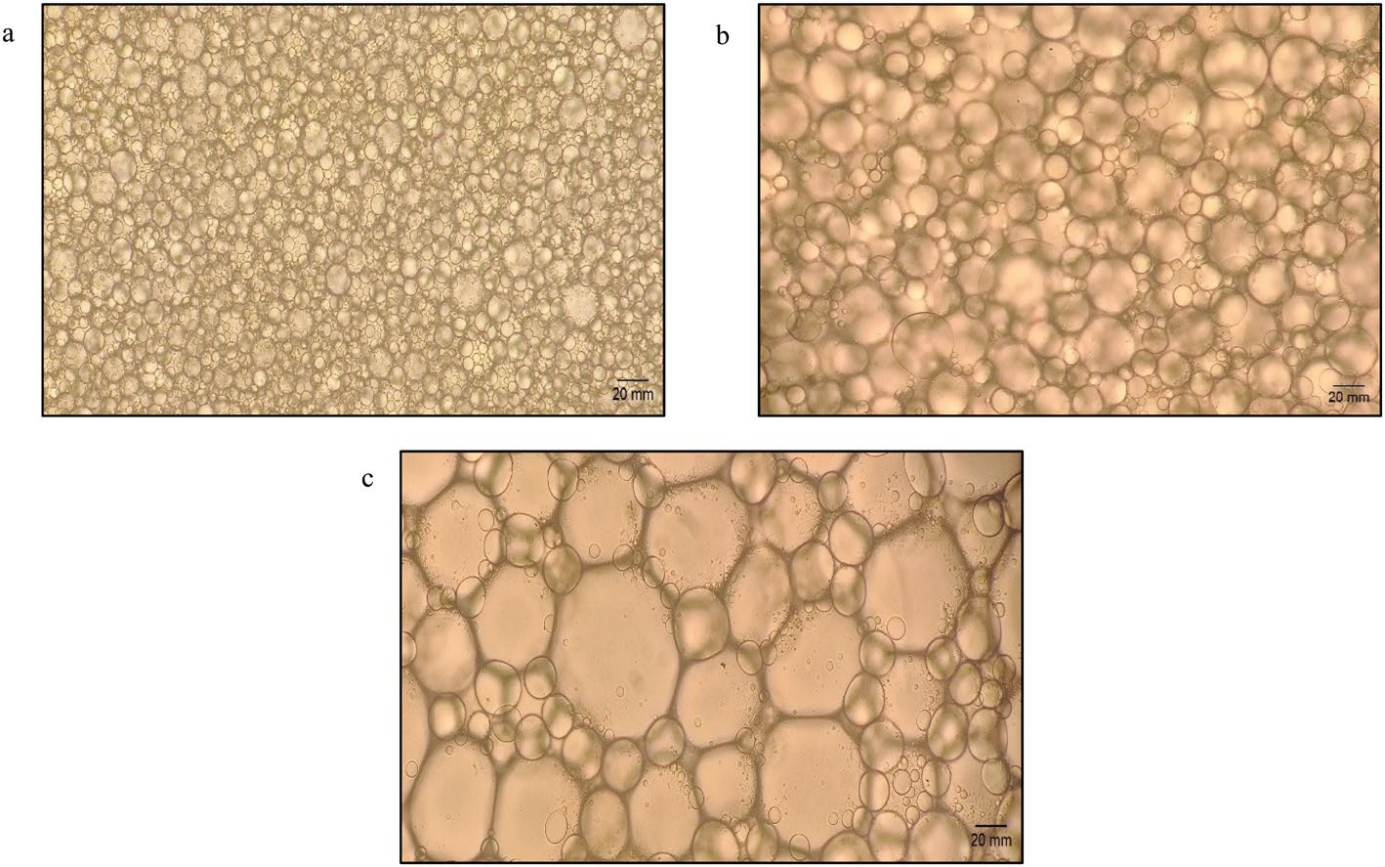
Droplet structure of mayonnaise emulsion at 20× magnification (a) egg yolk; (b) albumin; and (c) globulin.

The microscopic images of mayonnaise formulated using egg yolk, albumin, and globulin are shown in Figure 4. The parameters that determine the mayonnaise microstructure include the type of emulsifier, moisture content, and the manufacturing process. Both control and albumin mayonnaise exhibited an even distribution of round fat globules within the liquid phase. The small oil droplets in mayonnaise confer higher emulsion stability. In contrast, globulin mayonnaise exhibited a disrupted emulsion structure with large and small droplets.

### 3.7 Rheological Properties

**FIGURE 5:**
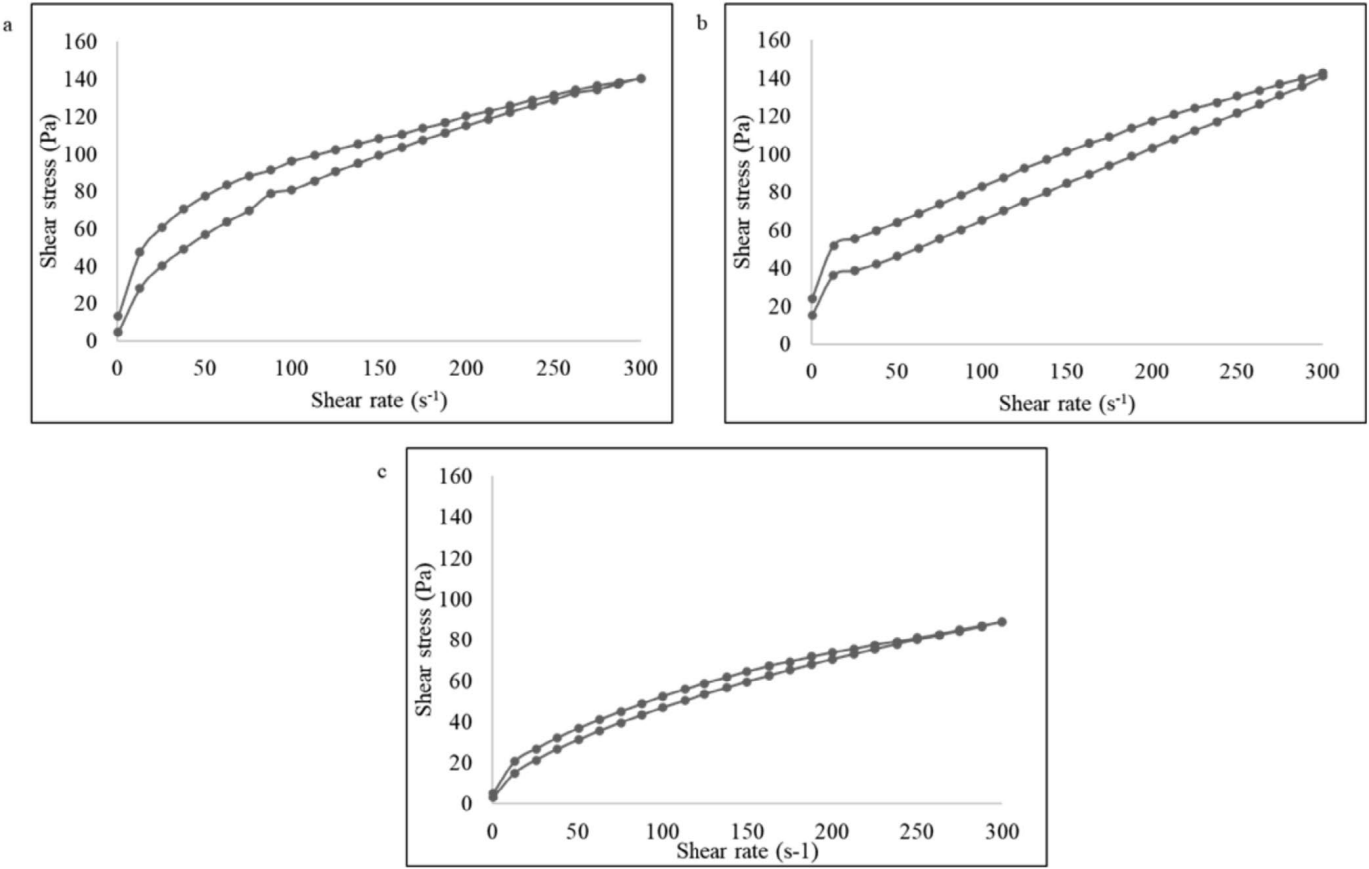
Flow curve of the mayonnaise sample (a) egg yolk; (b) albumin; (c) globulin. Data are presented as mean ± SD (*n* = 3).

The flow curve of control, albumin, and globulin mayonnaise is shown in Figure 5. The flow curve is defined as a plot of viscosity against shear rate, which helps in determining the critical shear rate (shear rate at which the viscosity starts to decrease), zero shear rate viscosity, rate of viscosity decrease with increasing/high shear rate, and yield stress (Janmey et al. 2007). Mayonnaise is a condiment that must be in a fluid state when dispersed and quickly restores to its slabby state to prevent flow out or spread. Thixotropy is a property in which structures progressively break down on shearing and slowly recover to their initial state at rest, depending on time and shear rate (Barnes 1997). All the mayonnaise samples in the current study exhibit thixotropic behavior over a range of shear rates (0–300 s^−1^). Thixotropic loops were obtained by linearly increasing, maintaining, and decreasing the shear rate.

Rheological models such as Power law, Herschel–Bulkley, and Carreau are used to test the fitness of mayonnaise flow behavior. The Herschel–Bulkley model is widely used to describe the non‐Newtonian fluid behavior of the sample with yield stress. The flow index (*n*), yield stress (*τ*_0_), and consistency (*K*) parameters of the Herschel–Bulkley model are presented in Table 3.

**TABLE 3:** Yield stress, flow index, consistency coefficient, and work of adhesion of vegan mayonnaise and control using the Herschel–Bulkley model.

Formulation	Yield stress *τ*_0_ (Pa)	Flow index *n* (−)	Consistency coefficient *K* (Pa.s^n^)	Work of adhesion (J/m^2^)
Egg yolk	0.4 ± 0.7	0.3 ± 0.0	19.7 ± 1.5	0.4 ± 0.2
Albumin	27.5 ± 2.7	0.6 ± 0.0	3.1 ± 1.0	0.4 ± 0.3
Globulin	1.4 ± 0.2	0.5 ± 0.0	5.2 ± 0.1	0.2 ± 0.1

*Note:* Data are presented as mean ± SD (*n* = 3).

Yield stress is defined as the minimum shear stress required to initiate the flow (Tadros 2013). The yield stress of albumin mayonnaise was higher than that of the control, while globulin mayonnaise marginally differed. Yield stress plays a key role in the stability of the emulsion system under low‐stress conditions, influencing storage, transportation, spreadability, mouthfeel (creaminess) during mastication, and processing steps such as homogenizing and pumping (Izidoro et al. 2008; Uribe‐Wandurraga et al. 2021; Schädle et al. 2022). Further, the increased yield stress is a favorable factor in determining the sample's ability to adhere to the food product (Mun et al. 2009), which was improved in albumin‐based mayonnaise.

The flow index (*n*) is an indicator to identify the fluid properties of the emulsion; *n* > 1, shear‐thickening fluid (or dilatant fluid); *n* = 1, Newtonian fluid; *n* < 1, shear‐thinning liquid. All the samples exhibit a shear‐thinning, pseudo‐plastic behavior with a flow index *n* < 1 (Table 3). Similar results were observed for mayonnaise prepared using pea pod powder, microalgae (low‐fat emulsion), soy milk with stabilizers (low‐fat emulsion), and traditional mayonnaise with different fat mimetics (Liu et al. 2007; Nikzade et al. 2012; Rudra et al. 2020; Uribe‐Wandurraga et al. 2021).

The consistency coefficient (*K*) of control was higher than that of albumin and globulin mayonnaise. A higher *K* value corresponds to a viscous emulsion, suggesting a strong structural network. The viscosity/*K* value of albumin and globulin mayonnaise might be increased by the addition of xanthan gum (Mun et al. 2009).

**FIGURE 6:**
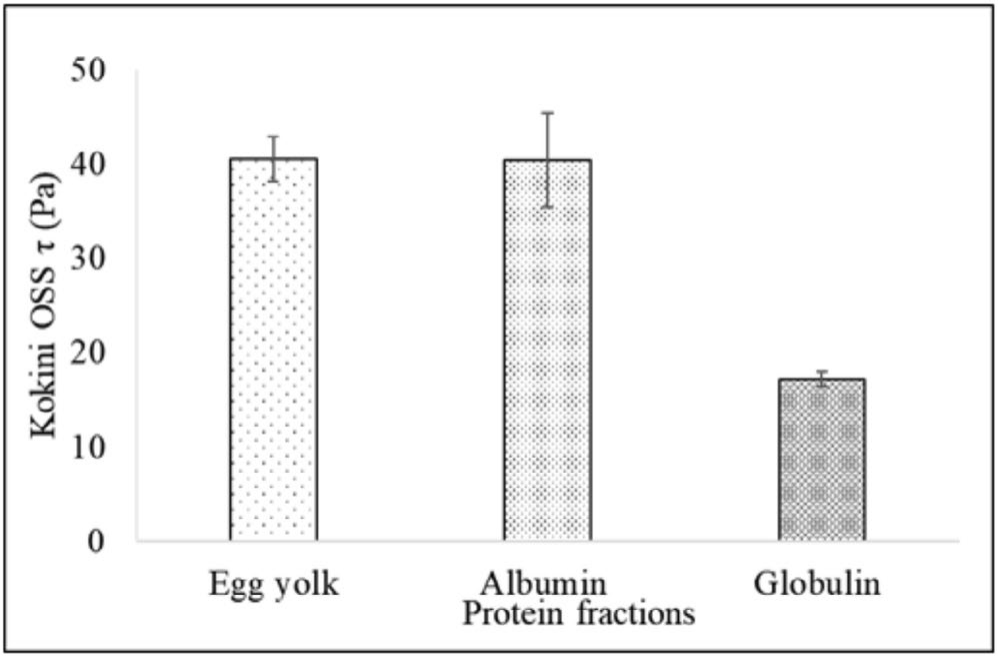
The Kokini OSS of vegan and traditional egg mayonnaise (control) formulations. Data are presented as mean ± SD (*n* = 3).

Kokini OSS was proposed by Kokini et al. (1978) to perceive the product's physical changes in the mouth during mastication. The yield stress, flow index, and consistency coefficient parameters necessary for Kokini OSS are obtained from the Herschel–Bulkley model. The Kokini OSS was lowest for globulin mayonnaise, while control and albumin mayonnaise had similar OSS (Figure 6). The lower Kokini OSS of globulin mayonnaise can be attributed to an increase in oil droplet diameter, resulting in viscosity loss (Katsaros et al. 2020).

The work of adhesion of samples is evaluated to determine mouthfeel and processability, as it also indicates the adherence of mayonnaise to the spreading knife. Both the control and albumin mayonnaise exhibited similar work of adhesion, suggesting a comparable mouthfeel and spreading characteristics (Table 3). In contrast, the globulin mayonnaise displayed negligible work of adhesion. Additionally, results from the probe tack test indicate that the adhesive profile of albumin mayonnaise closely matched that of the control, further supporting albumin mayonnaise as the closest match.

The mayonnaise made with glutelin is not characterized in detail in this study due to its high viscosity, inconsistent yield stress, and consistency coefficient. The high viscosity and inconsistent yield stress of mayonnaise are related to its pH (4.2–4.3), which coincides with the average isoelectric point (pI) of the proteins. When approaching the pI, the charge carried on the protein is reduced to nearly zero, decreasing the electrostatic repulsion between the droplets and leading to aggregation (Bai and McClements 2016; Li et al. 2022). This increases friction, leading to a higher viscosity and inconsistent yield stress (Li et al. 2022). Glutelin mayonnaise exhibited a lower thermal stability than the other mayonnaise samples and the control. The results of the work of adhesion also suggest that the sample is more adhesive, and more work is required to rebind to the surface. Further, oil exudation was observed due to the disruption of the emulsion structure at elevated temperatures.

## 4 Conclusion

This study demonstrates the potential of plant‐based protein extracted from wheat bran as an alternative to egg‐based emulsifiers in vegan mayonnaise. Among the fractions, albumin showed comparable performance to the control in terms of stability and functional properties, indicating its suitability for emulsion‐based applications. The findings highlight the value of utilizing wheat bran, a by‐product of milling, as a sustainable ingredient in food formulations. Overall, the results support the feasibility of developing stable, plant‐based mayonnaise without compromising key quality attributes. Future work should focus on evaluating long‐term storage stability and sensory characteristics to further assess consumer acceptance.

## Author Contributions

**Varshini Krishnamoorthy:** conceptualization, investigation, methodology, writing – original draft, visualization, formal analysis. **Abinaya Arunachalam:** investigation, writing – review and editing. **Jan Krijgsheld:** conceptualization, writing – review and editing, formal analysis, supervision, validation. **Gert‐Jan W. Euverink:** conceptualization, writing – review and editing, formal analysis, project administration, supervision, resources, validation, funding acquisition, data curation.

## Funding

This work was supported by Samenwerkingsverband Noord‐Nederland (R010).

## Conflicts of Interest

The authors declare no conflicts of interest.

## Data Availability

The data that support the findings of this study are available from the corresponding author upon reasonable request.
